# Is Distraction an Adaptive or Maladaptive Strategy for Emotion Regulation? A Person-Oriented Approach

**DOI:** 10.1007/s10862-016-9570-x

**Published:** 2016-09-07

**Authors:** Martin Wolgast, Lars-Gunnar Lundh

**Affiliations:** Department of Psychology, Lund University, Box 213, 221 00 Lund, Sweden

**Keywords:** Distraction, Acceptance, Avoidance, Emotion regulation, Cognitive restructuring, Positive emotionality, Negative emotionality, Quality of life, Cluster analysis

## Abstract

Distraction is an emotion regulation strategy that has an ambiguous status within cognitive-behavior therapy. According to some treatment protocols it is counterproductive, whereas according to other protocols it is seen as a quite useful strategy. The main purpose of the present study was to test the hypothesis that distraction is adaptive when combined with active acceptance, but maladaptive when combined with avoidant strategies. A non-clinical community sample of adults (*N* = 638) and a clinical sample (*N* = 172) completed measures of emotion regulation and well-being. Hierarchical cluster analysis was used to identify subgroups with different profiles on six emotion regulation variables, and these subgroups were then compared on well-being (positive and negative emotionality, and life quality) and on clinical status. A nine-cluster solution was chosen on the basis of explained variance and homogeneity coefficients. Two of these clusters had almost identical scores on distraction, but showed otherwise very different profiles (distraction combined with acceptance vs. distraction combined with avoidance). The distraction-acceptance cluster scored significantly higher than the distraction-avoidance cluster on all measures of well-being; it was also under-represented in the clinical sample, whereas the distraction-avoidance cluster was over-represented. Limitations include a cross-sectional design, and use of self-report measures. The findings suggest that distraction may be either adaptive or maladaptive, depending on whether it is combined with an attitude of acceptance or avoidance.

Difficulties in emotion regulation are increasingly being incorporated into models of psychopathology, and are the direct target of treatment interventions in several recent treatment protocols (Gratz and Tull 2011; Barlow et al. 2011; Berking et al. 2008; Linehan 1993a; Lynch et al. 2007).

An important question in this area concerns the usefulness of strategies focusing on changing versus accepting emotional experiences (e.g. Hayes et al. 1999; Mathews 2006; Clark 1999; Arch and Craske 2008; Hayes 2008; Hofmann and Asmundson 2008). On the one hand, models emphasizing the value of cognitive change strategies are consistent with evidence that the emotional reactions of humans depend to a considerable extent on the way situations or experiences are cognitively construed or interpreted (e.g. Murphy and Zajonc 1993; LeDoux 1993; Russel 2003). From the perspective of Acceptance and Commitment Therapy (ACT; Hayes et al. 1999) on the other hand, efforts to change or otherwise control emotions or thoughts run the risk of maintaining an inner struggle with these events (Hayes 2008), that leads to suffering and impaired functioning (Hayes et al. 1999). Instead, in ACT, the focus is on learning to observe and accept dysfunctional emotional experiences, in order to become free of this inner struggle.

An important part of the theoretical discussion regarding the relative value of cognitive change and acceptance strategies concern how these approaches or concepts relate to each other. In the discussion regarding these matters, some writers have proposed that acceptance and cognitive change strategies show considerable similarities and achieve similar outcomes (Arch and Craske 2008), whereas others see them as distinct strategies or approaches that are mutually exclusive (Blackledge and Hayes 2001), and still others view them as different but compatible forms of emotion regulation intervening at different points in the emotion generating process (Hofmann and Asmundson 2008).

In a previous study, Wolgast et al. (2012) explored the constructs of cognitive restructuring and acceptance by using items from well-established measures of these respective constructs in order to identify subcategories or conceptual nuances in this area. Exploratory factor analyses in a non-clinical sample resulted in a six-factor solution. Three of these factors (“Active Acceptance”, “Thought Avoidance” and “Resignation”) loaded on a higher order factor of “Acceptance”, whereas three other factors (“Constructive Refocusing”, “Cognitive Reappraisal” and “Distractive Refocusing”) loaded on a higher order factor of “Cognitive Restructuring”. The factors are described further below in the “methods” section. Interestingly, “Active Acceptance” also loaded significantly on the “Cognitive Restructuring” factor, whereas “Constructive Refocusing” also loaded on the “Acceptance” factor. This factor structure was validated by confirmatory factor analyses in another non-clinical sample and in a clinical sample. In sum, these findings indicate that acceptance and cognitive restructuring should not be regarded as unitary and non-related constructs, but rather as partly overlapping general dimensions of emotion regulation consisting of several sub-constructs.

In studying these six emotion regulation factors for their associations with well-being (positive and negative emotionality, life quality, and clinical status), Wolgast et al. (2012) found that Constructive Refocusing was most consistently positively associated with aspects of well-being, but that also Active Acceptance and Cognitive Reappraisal showed positive associations of this kind. Thought Avoidance and Resignation, as expected, showed consistent negative associations with all aspects of well-being. The strategy of Distractive Refocusing, however, stood out as not showing any significant associations with positive or negative emotionality, or with life quality, and showing only a weak association with clinical status (the non-clinical group using more distractive refocusing than the clinical group).

These results with regard to Distractive Refocusing are interesting in view of the ambiguous status of distraction strategies within cognitive behavioral therapies. In some protocols, distraction is seen as counter-productive (e.g. Craske and Barlow 2008). In other protocols, however, as for example in Dialectic Behavior Therapy (DBT; Linehan 1993a) and in Gratz’ (Gratz and Tull 2011) Emotion Regulation Group Treatment (ERGT), distraction is taught as a valid strategy for regulating aversive emotions. One possibility is that distraction is adaptive in some contexts, and maladaptive in others. If so, this might also explain the weak associations between Distractive Refocusing and well-being in our previous study (Wolgast et al. 2012), when measured as a variable averaged over different context, based on questions about what the respondents *generally* do or think when faced with aversive emotions. An alternative possible explanation for these weak associations, however, is that Distractive Refocusing simply represents an emotion regulation strategy that is neither particularly effective nor maladaptive.

In connection to this, it is interesting to note that when distraction is taught in DBT (Linehan 1993b) or ERGT (Gratz 2010), it is done in a context which strongly emphasizes the importance of *acceptance* and *willingness* in relation to aversive experiences. For example, as Linehan (1993b) teaches distraction to clients, it is done as an example of a *distress tolerance* skill that may be important for survival in situations of crisis. As Gratz (2010) describes it, distraction involves redirecting attention towards something else *for a short period of time*, and is therefore quite compatible with a willingness to come into contact with the avoided emotion in the near future. As she puts it, *if distraction is used in this way*, it can be a useful strategy for taking the edge off painful emotions.

This suggests the hypothesis that distraction is an adaptive strategy if it is used *in the context of an accepting attitude* to painful emotions, whereas it is maladaptive if used *in the context of experiential avoidance*. That is, distraction would be adaptive if it is only a matter of temporarily redirecting one’s attention toward something else for a short period of time, with the intention of getting back into contact with that emotion or the challenging situation in the near future. On the other hand, if distraction is used as part of a generally avoidant strategy, it is likely to be maladaptive. The main purpose of the present study was to test this hypothesis by comparing the well-being of (1) individuals who use distraction in combination with acceptance and (2) individuals who use distraction in combination with experiential avoidance. To test this hypothesis, we used a person-oriented approach to reanalyze the data that were analyzed by a variable-oriented approach in the study by Wolgast et al. (2012), which we believe is justified given that we use an entirely different approach to data analysis, test distinctly different hypotheses and present completely novel results compared to the previous study.

A general limitation of the kind of variable-oriented approach that was used in the previous study is that it tells us nothing about *how different strategies are combined at the level of the individual.* This issue is of theoretical importance, among other things, because it bears on the question of whether different strategies (e.g., cognitive restructuring strategies and acceptance strategies) are readily combined or contradictory. In addition, by identifying subgroups of individual who share the same profile of scores on the emotion regulation variables we may not only see how these different strategies are combined in different individuals and how frequent such combinations are, but also how these different profiles or patterns relate to measures of psychological well-being. A second purpose of the present study was therefore to explore these issues more generally, by contrasting alternative hypotheses about the effects of combining acceptance and cognitive restructuring strategies, and the effects of lacking these emotion regulation strategies.

Based on the theoretical discussion summarized above, we expected to find one subgroup of individuals who rely on acceptance strategies but not on cognitive restructuring, another subgroup of individuals who rely on cognitive restructuring but not on acceptance, and a third subgroup of individuals who use both cognitive restructuring and acceptance. As to their association with psychological well-being, we may contrast three hypotheses: (1) *The equifinality hypothesis*: If acceptance and cognitive change strategies represent strategies with considerable similarities that achieve similar outcomes ( e.g., Arch and Craske 2008), all three subgroups should be equally high on psychological well-being, and be overrepresented in the non-clinical sample. (2) *The additive/interactive hypothesis:* If acceptance and cognitive restructuring are different but compatible forms of emotion regulation intervening at different points in the emotion generative process (e.g., Hofmann and Asmundson 2008), the effects of these different strategies may be expected to add or interact positively so that the subgroup of individuals who are high on both would score higher than those who are high on only one of these strategies. (3) *The “acceptance-is-essential” hypothesis:* If acceptance is an underlying functional dimension that is basic to all adaptive strategies of emotion regulation (Blackledge and Hayes 2001; Boulanger et al. 2010), subgroups who score generally high on acceptance should show the highest psychological well-being, regardless of their scores on cognitive restructuring.

Similarly, at the other end of the scale, we might expect to find one subgroup of individuals who score generally *low* on acceptance but not on cognitive restructuring, another subgroup of individuals who score generally low on cognitive restructuring but not on acceptance, and a third subgroup with low scores on both cognitive restructuring and acceptance. Again, alternative hypotheses may be contrasted: (1) *The “no-strategies-is-worst” hypothesis*: If both acceptance and cognitive change strategies are adaptive strategies, a subgroup with low scores on both of these strategies should be worst off in terms of psychological well-being. (2) *The “acceptance-is-essential” hypothesis:* If acceptance is an underlying functional dimension that is basic to all adaptive strategies of emotion regulation (Blackledge and Hayes 2001; Boulanger et al. 2010), individuals who score generally low on acceptance should be worst off, whether they score low on cognitive restructuring or not.

To summarize, the present research had two purposes. The first purpose was to test the hypothesis that the use of distractive strategies is adaptive when combined with acceptance, and maladaptive when combined with avoidance. The second purpose was to compare alternative hypotheses with regard to the how strategies related to cognitive restructuring and acceptance may be combined, as well as how different combinations relate to measures of psychological well-being. In order to test these hypotheses we used a person oriented approach to data analysis which means that we studied if it was possible to identify groups of individuals with similar profiles of scores across the studied variables and examine how these different groups scored on measures of psychological well-being.

## Method

### Participants

Informed consent was obtained from all individual participants included in the study.

#### Non-Clinical Sample

A non-clinical sample of 1500 individuals (aged 18-70) was drawn randomly from the SPAR register (the Swedish government´ Person and Address Register), and were sent a letter with a questionnaire and a pre-stamped addressed return envelope. Of these, 638 individuals (364 women and 274 men, response rate 42 %) filled out the entire questionnaire and returned it. The letter also included information regarding the study as well as the measures. Participation was anonymous and no information was stored that could identify a specific participant. In addition to the measures to be used in the study, the participants were asked to state their gender, age and level of highest completed education.

#### Clinical Sample

Participants in the clinical sample (*N* = 172) were volunteers recruited among patients currently in treatment in open psychiatric care in the county of Blekinge in Sweden. In total 350 booklets containing an information letter and all the measures were given to members of staff in open psychiatric care, who in turn administered them to clients they were in contact with. Of these 350 booklets, 172 were returned, rendering a response rate of 49 %. Of the respondents, 63 % were female and 37 % were male. There were no formal exclusionary criteria to participate in the study and we had no means of controlling who were asked to participate and who volunteered, nor their diagnosis and type of treatment. The population from which the sample was drawn (patients attending open psychiatric care in Blekinge during 2011) are known to have the following characteristics: 43 % are male, 57 % are female and the most common diagnostic groups are anxiety disorders (15 %), depressive disorders (14 %), schizophrenia and other psychotic disorders (13 %), personality disorders (9 %), bipolar disorder (7 %), neuropsychiatric disorders including mental retardations (7 %), substance abuse (5 %) and post-traumatic stress disorder (5 %).

### Measures

#### Strategies of Emotion Regulation

To assess different types of strategies we used the participants’ scores on scales based on the factors identified in a previous study (Wolgast et al. 2012). There were three acceptance-related factors: *Thought Avoidance* (14 items), which refers to active efforts to avoid and suppress aversive cognitive material, *Active Acceptance* (7 items), which represents a combination of experiential acceptance and behavioral flexibility in the face of aversive emotions, and *Resignation* (4 items), which refers to passively accepting a situation or an aversive emotional state combined with an experience of not having the ability to do anything about it. There were also three factors related to Cognitive Restructuring: *Cognitive Reappraisal* (6 items), which refers to the traditional concept of reappraisal (i.e. changing emotional reactions by changing our appraisals of the emotion eliciting stimulus or situation), *Distractive Refocusing* (6 items), which represents strategies aimed at trying to think about something else, preferably something positive, entailing an unwillingness to remain in cognitive contact with the emotion eliciting stimulus or situation rather than to think differently about it, and finally *Constructive Refocusing* (12 items)*,* which refers to attempts to change not how we interpret the topography of the situation (i.e. how we interpret the factual characteristics of the events) but rather to reframe or reinterpret the function or consequence of the situation (e.g. what our behavioral options are given what has happened, what we can learn from the situation etc). All the scales showed adequate levels of internal consistency (Cronbach’s alphas: Thought Avoidance: .92, Active Acceptance: .75, Resignation: .73, Cognitive Reappraisal: .84, Distractive Refocusing: .79, Constructive Refocusing: .87).

#### Positive and Negative Affect Scale (PANAS)

To assess dispositional positive and negative emotionality participants completed the Positive and Negative Affect Schedule (PANAS; Watson et al. 1988). The PANAS is a 20-item mood adjective checklist designed to measure the Positive Affect (PA) and Negative Affect (NA) factors and has shown satisfactory psychometric properties in previous research (Watson et al. 1988). To complete the PANAS, participants were instructed to use a five-point Likert scale (1, very slightly or not at all; 5, extremely) to indicate “to what extent you generally feel this way, that is, how you feel on the average” for each adjective. The Swedish version of the scale showed adequate internal consistency for both Positive Affect and Negative Affect (Cronbach’s alphas: Positive Affect = .89; Negative Affect = .92).

#### World Health Organization Quality of Life Assessment—Brief Version (WHOQOL-BREF)

Quality of life was assessed by the WHOQOL-BREF, developed and validated by WHO in several studies (Skevington et al. 2004). It contains 26 items, the response (from least to most) to each item being on a 5-point rating scale of a particular aspect of quality of life. Besides the first two items of general nature, the remaining 24 items of the instrument are known to factorise into four domains of quality of life, denoted by ‘physical health’ (7 items, domain 1), ‘psychological’ (6 items, domain 2), ‘social relationships’ (3 items, domain 3), and ‘environment’ (8 items, domain 4), respectively. The Swedish version of the scale used in the present study showed adequate internal consistency (Cronbach’s alpha = .87).

### Hierarchical Cluster Analysis

The individual participants were grouped into clusters on the basis of their individual profiles of scores on the six scales related to acceptance and cognitive restructuring. This was done according to the LICUR procedure suggested by Bergman (1998) and using the statistical package for pattern-oriented analyses SLEIPNER 2.1 (Bergman and El-Khouri 2002). In a first step, the data were searched for multivariate outliers, which led to the identification and removal of one outlier. Secondly, clusters were formed using Ward’s hierarchical clustering method, which is a stepwise procedure that starts by considering each individual as a separate cluster and then merges the two clusters that results in the smallest increase in the overall error sum of squares in each subsequent step. In order to determine the optimal cluster solution we used the criteria suggested by Bergman (1998): (1) The cluster solution should be theoretically meaningful. (2) If a distinct reduction in the explained variance occurs when moving from one step to another, this may indicate that two not so similar clusters have been merged and that the resulting cluster solution is not optimal. (3) The number of clusters should not be expected to be less than five or to exceed fifteen. (4) The size of the explained variance for the chosen cluster solution should at the very least exceed 50 %, and preferably exceed 67 %. Additionally, the homogeneity coefficients of each cluster should preferably be <1.0. In a third step, a data simulation using Monte Carlo procedure with 20 re-samplings was performed to test if the explained variance for the identified cluster solution significantly exceeded what would be expected from a random data set with the same general properties as the “real” data set. In a final step, in order to improve the homogeneity of the clusters and to increase the proportion of explained variance, a non-hierarchical relocation procedure was performed (Bergman 1998), in which individuals were moved between clusters in order to find the optimal solution.

After removing an outlier identified by the residue procedure in SLEIPNER 2.1 (Bergman and El-Khouri 2002), 809 individuals were left for further analyses using Ward’s hierarchical clustering method. Applying the criteria suggested by Bergman (1998) resulted in the choice of a 9-cluster solution, which explained 59.9 % of the variance, whereas the 8 cluster solution would have resulted in a drop in explained variance to 57.1 %. The data simulation reliably showed that the explained variance for the chosen cluster solution was significantly higher than what would be expected by chance (*p* < .01). After this, the relocation procedure was performed, resulting in a final nine cluster solution which explained 62.6 % of the variance and where all the clusters except one had homogeneity coefficients of <1.0 (cluster 5 had a homogeneity coefficient of 1.08).

## Results

First, descriptive data is presented on the two samples. Then the results of the cluster analysis are described, and the clusters are compared on positive and negative emotionality, life quality and clinical status. After that, the results are analyzed with regard to the research questions: First the hypothesis on distraction is tested. Second, the alternative hypotheses concerning the combination of a high use, and low use, of both acceptance and cognitive restructuring are compared.

### Descriptive Data

Descriptive data on demographic and clinical variables for the clinical and non-clinical samples are presented in Table 1. On the demographic variables, the samples differed significantly with regard to age, *t* (808) = 4.54, *p* < .01, and level of education, χ^2^ (2) = 11.4, *p* < .01, but not with regard to gender, χ^2^ (1) = 1.54, *p* = .21. With regard to positive and negative emotionality and quality of life, the differences between the samples were significant and large for all variables [PANAS-N: *t* (808) = 15.9, *p* < .01, *d* = 1.1; PANAS-P: *t* (808) = -17.1, *p* < .01, *d* = -1.2; Total WHOQOL: *t* (808) = 19.0, *p* < .01, *d* = 1.3]. The bivariate correlations between all self-report measures are presented in Table 2.

**Table 1:** Descriptive statistics for demographic and psychological variables

Variable	Non-clinical sample (*N* = 638)	Clinical sample (*N* = 172)
	*%*	*%*
Gender		
*Men*	43	37
*Women*	57	63
Highest education		
*Elementary school*	18.4	26.3
*Gymnasium*	47.0	53.4
*University*	34.6	20.3
	*M (SD)*	*M (SD)*
Age	43.5 (14.7)	37.8 (12.8)
Psychological variables		
Negative emotionality (*PANAS-N*)	20.4 (7.6)	31.7 (7.8)
Positive emotionality (*PANAS-P*)	35.2 (6.3)	26.1 (6.9)
Quality of Life (*WHOQOL*)	98.0 (15.3)	72.9 (15.8)

**Table 2:** Bivariate correlations (Pearson’s) between all self-report measures (*N* = 809)

	PANAS N	WHOQOL	Thought Av.	Active Acc.	Resig.	Constr. Ref.	Cog. Reap.	Distr. Ref.
PANAS P	–.53^*^	.65^*^	–.54^*^	.51^*^	–.56^*^	.57^*^	.51^*^	.45^*^
PANAS N		–.69^*^	.68^*^	–.58^*^	.65^*^	–.42^*^	–.26^*^	–.20^*^
WHOQOL			–.65^*^	.62^*^	–.63^*^	.52^*^	.37^*^	.23^*^
Thought Av.				-.58^*^	.64^*^	–.35^*^	–.11^*^	–.09^*^
Active Acc.					–.46^*^	.49^*^	.34^*^	.26^*^
Resig.						–.31^*^	–.16^*^	–.08^*^
Constr. Ref.							.51^*^	.45^*^
Cog. Reap.								.39^*^

^*^ *p* < .001

### Cluster Analysis

**Fig. 1:**
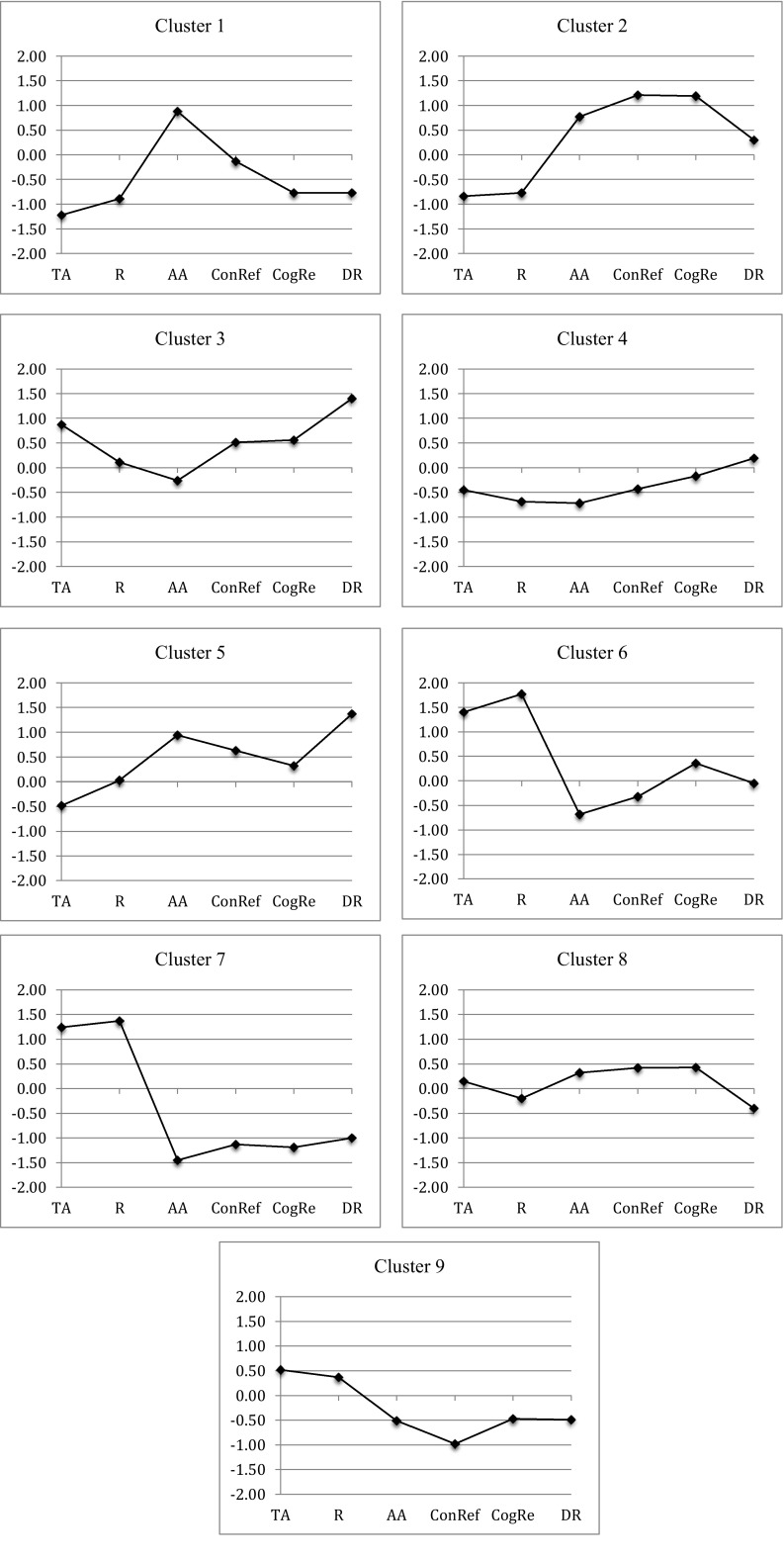
Pattern of z-scores across the 6 factors for all clusters. *TA* thought avoidance, *R* resignation, *AA* active acceptance, *ConRef* constructive refocusing, *CogRe* cognitive reappraisal, *DR* distractive refocusing

As stated above (see the section on hierarchical cluster analysis), the clustering procedure resulted in a nine cluster solution. Table 3 shows the means and standard deviations on each factor for each cluster as well for the whole group, whereas Fig. 1 displays the profile of *z*-scores (cluster mean minus total group mean, divided by the standard deviation of the total group) for each cluster on the 6 factors.

**Table 3:** The nine-cluster solution, with scores on the six emotion regulation variables

Cluster	*N*	Thought avoidance *M (SD)*	Active acceptance *M (SD)*	Resignation *M (SD)*	Constructive refocusing *M (SD)*	Cognitive reappraisal *M (SD)*	Distractive refocusing *M (SD)*
1	104	34.0 (8.8)	21.6 (2.7)	6.7 (1.6)	30.7 (7.6)	19.0 (6.1)	10.5 (2.6)
2	108	40.1 (10.9)	21.0 (2.5)	7.1 (1.9)	43.4 (4.3)	33.7 (4.3)	15.1 (2.8)
3	62	67.3 (8.31)	16.2 (2.7)	10.2 (2.0)	36.7 (6.8)	29.0 (4.4)	19.8 (3.2)
4	83	46.4 (9.0)	14.0 (2.5)	7.4 (1.8)	27.8 (6.3)	23.5 (5.0)	14.6 (2.8)
5	94	45.8 (8.9)	21.8 (2.9)	9.9 (3.1)	37.9 (6.5)	27.2 (6.8)	19.7 (2.9)
6	48	75.7 (6.8)	14.2 (3.5)	16.0 (2.0)	28.9 (6.7)	27.5 (4.7)	13.6 (2.7)
7	87	73.2 (6.7)	10.6 (3.1)	14.6 (2.1)	21.2 (7.2)	15.9 (5.0)	9.5 (2.1)
8	112	55.9 (9.1)	18.9 (2.7)	9.1 (2.0)	35.9 (5.5)	28.0 (4.5)	12.1 (1.9)
9	111	61.7 (7.6)	15.0 (2.7)	11.1 (1.8)	22.6 (3.8)	21.3 (5.3)	11.7 (2.4)
All	809	53.5 (15.9)	17.4 (4.7)	9.8 (3.5)	31.9 (9.5)	24.8 (7.5)	13.8 (4.3)

### Comparisons Between the Clusters on Well-Being

A one-way ANOVA with the nine clusters as independent variable and age as dependent variable showed a significant omnibus effect [*F*(8, 800) = 2.74, *p* < .01]. Tukey-corrected post-hoc comparisons showed that the only significant differences between the clusters were that Cluster 4 was older than Cluster 3 and Cluster 7. The gender distribution did not differ significantly across the nine clusters [*χ* ^2^(8) = 13.9, *p* = .09]. With regard to level of highest completed education, there was a significant omnibus effect, indicating that there were significant differences in the distribution across the clusters [*χ* ^2^(16) = 46.2, *p* < .01]. To further investigate this effect, the observed frequency in each cell was compared with the frequency expected if the educational level was randomly distributed across the clusters. The statistical testing was performed in accordance with the fixed-margins model using EXACON (Bergman and El-Khouri 1987). After adjusting the alpha level to allow for multiple comparisons using Bonferroni correction (critical α = .002; .05/27) the analysis showed that the only significant effect was that more participants in Cluster 2 had completed a university level education than what was to be expected by chance (observed frequency 54, expected frequency 34.2, *χ* ^2^ = 11.5, *p* = .0008).

Three Bonferroni corrected (critical α’s = .017) ANOVAs were performed with the nine clusters as independent variables and scores on PANAS-N, PANAS-P and WHOQOL as dependent variable in the separate analyses. To control for the observed differences between the clusters with regard to age and level of highest completed education, these variables were entered as covariates in the analyses. Means and standard deviations on the criterion variables for each cluster are presented, in descending order according to the mean score of the clusters, in Tables 3, 4 and 5 (one table for each criterion variable). The omnibus tests showed that there were significant differences between the clusters on all three variables [PANAS-P: *F*(8, 798) = 85.1, *p* < .01, partial η^2^ = .46; PANAS-N: *F*(8798) = 116.9, *p* < .01, partial η^2^ = .54; WHOQOL: *F*(8, 798) = 110.7, *p* < .01, partial η^2^ = .53]. Sidak-corrected post hoc comparisons were performed to examine the significance of the differences between the clusters on each dependent variable. The results from these analyses are presented in Tables 3, 4 and 5.

**Table 4:** Comparisons between the clusters on the PANAS-P

Cluster	*N*	PANAS-P *M (SD)*	Significant differences (Sidak, α = .05)
2. Generally High Acceptance + High Cognitive Restructuring (except Distractive Refocusing)	108	39.4 (3.8)	> Clusters 4, 8, 3, 6, 9, 7
1. Generally High Acceptance + Low Cognitive Restructuring (except Constructive Refocusing)	104	37.7 (4.4)	> Clusters 8, 3, 6, 9, 7
5. High Distractive Refocusing + High Active Acceptance	94	37.1 (5.2)	> Clusters 3, 6, 9, 7
4. Low Active Acceptance + Low Resignation	83	35.2 (5.4)	> Clusters 6, 9, 7
8. Average profile (neither high nor low on any factor)	112	34.5 (6.6)	> Clusters 6, 9, 7
3. High Distractive Refocusing + High Thought Avoidance	62	33.4 (6.4)	> Clusters 6, 9, 7
6. Generally Low Acceptance + Average Cognitive Restructuring	48	28.6 (6.8)	> Cluster 7
9. Low Constructive Refocusing	111	27.8 (5.9)	> Cluster 7
7. Generally Low Acceptance + Generally Low Cognitive Restructuring	87	22.6 (6.2)	
All	809	33.3 (7.7)	

“High”: *z* > .70; “Low”: *z* < -.70

**Table 5:** Comparisons between the clusters on the PANAS-N

Cluster	*N*	PANAS-N *M (SD)*	Significant differences (Sidak, α = .05)
7. Generally Low Acceptance + Generally Low Cognitive Restructuring	87	35.8 (6.1)	> Clusters 9, 3, 8, 4, 5, 1, 2
6. Generally Low Acceptance + Average Cognitive Restructuring	48	34.9 (7.5)	> Clusters 9, 3, 8, 4, 5, 1, 2
9. Low Constructive Refocusing	111	26.6 (6.2)	> Clusters 8, 4, 5, 1, 2
3. High Distractive Refocusing + High Thought Avoidance	62	25.1 (8.7)	> Clusters 8, 4, 5, 1, 2
8. Average profile (neither high nor low on any factor)	112	20.1 (6.0)	> Clusters 1, 2
4. Low Active Acceptance + Low Resignation	83	19.8 (5.4)	> Clusters 1, 2
5. High Distractive Refocusing + High Active Acceptance	94	19.1 (5.3)	> Clusters 1, 2
1. Generally High Acceptance + Low Cognitive Restructuring (except Constructive Refocusing)	104	16.4 (5.0)	
2. Generally High Acceptance + High Cognitive Restructuring (except Distractive Refocusing)	108	16.2 (5.0)	
All	809	22.8 (8.9)	

“High”: *z* > .70; “Low”: *z* < -.70

### Representation of the Clusters in the Clinical and non-Clinical Samples

To study the over- and under-representation of each cluster in the clinical and non-clinical samples, the clusters were cross-tabulated with the two samples. Table 7 shows a comparison of the observed frequencies in each cell with the frequencies expected if the clusters had been randomly distributed across the samples. The statistical testing was performed in accordance with the fixed-margins model using EXACON (Bergman and El-Khouri 1987).

### The Adaptiveness and Maladaptiveness of Distractive Refocusing

As seen in Table 3 and in Fig. 1, two clusters showed high scores on Distractive Refocusing: cluster 3 and cluster 5. These two clusters had almost exactly the same score on Distractive Refocusing, but showed otherwise very different profiles. Whereas Cluster 3 combined high scores on Distractive Focusing with high scores on Thought Avoidance, Cluster 5 combined high scores on Distractive Refocusing with high scores on Active Acceptance. According to the hypothesis, Cluster 5 should show higher well-being than Cluster 3. This hypothesis was supported on all four variables: Cluster 5 scored significantly higher than Cluster 3 on positive emotionality (see Table 3), significantly lower on negative emotionality (see Table 4), and significantly higher on life quality (see Table 5). In addition, as seen in Table 6, Cluster 5 was under-represented in the clinical sample (observed frequency: 11, expected frequency: 20, *χ* ^2^ = 4.0, *p* < .05), whereas Cluster 3 was over-represented in the clinical sample (observed frequency: 20, expected frequency: 13, *χ* ^2^ = 3.6, *p* < .05).

**Table 6:** Comparisons between the clusters on the WHOQOL

Cluster	*N*	WHOQOL *M (SD)*	Significant differences (Sidak, α = .05)
2. Generally High Acceptance + High Cognitive Restructuring (except Distractive Refocusing)	108	107.2 (9.1)	> Clusters 5, 8, 4, 3, 9, 6, 7
1. Generally High Acceptance + Low Cognitive Restructuring (except Constructive Refocusing)	104	106.2 (9.7)	> Clusters 8, 4, 3, 9, 6, 7
5. High Distractive Refocusing + High Active Acceptance	94	101.7 (14.0)	> Clusters 3, 9, 6, 7
8. Average profile (neither high nor low on any factor)	112	98.0 (13.1)	> Clusters 3, 9, 6, 7
4. Low Active Acceptance + Low Resignation	83	96.4 (16.1)	> Clusters 9, 6, 7
3. High Distractive Refocusing + High Thought Avoidance	62	91.1 (15.9)	> Clusters 9, 6, 7
9. Low Constructive Refocusing	111	79.8 (12.6)	> Clusters 6, 7
6. Generally Low Acceptance + Average Cognitive Restructuring	48	72.0 (15.1)	
7. Generally Low Acceptance + Generally Low Cognitive Restructuring	87	67.3 (9.9)	
All	809	92.7 (18.5)	

“High”: *z* > .70; “Low”: *z* < -.70

### Combining High Use of Acceptance with High Use of Cognitive Restructuring

As seen in Fig. 1, one of the clusters (Cluster 1) was characterized by consistently high scores on acceptance and consistently low scores on cognitive restructuring, and another (Cluster 2) by consistently high scores on both acceptance and cognitive restructuring. Interestingly, these two clusters showed a very similar profile on the acceptance factors, but differed widely on the cognitive restructuring factors. Still, as seen in Tables 4, 5, 6 and 7, they did not differ significantly on any of the indicators of well-being. Interestingly, although both of these clusters were large (104 individuals in Cluster 1, and 108 individuals in Cluster 2), they were both completely unrepresented in the clinical sample – all 212 individuals were from the non-clinical sample. This speaks against the hypothesis that acceptance and cognitive restructuring have an additive or interactive effect on well-being. The results are, however, consistent with both of the other hypotheses – that is, the equifinality hypothesis (i.e., acceptance and cognitive change strategies achieve similar outcomes), and the “acceptance-is-essential hypothesis”. Unfortunately, the cluster analysis did not identify any cluster with consistently high scores on cognitive restructuring and consistently low scores on acceptance – if so, it would have been possible to contrast these two hypotheses.

**Table 7:** Cross-tabulation of samples and clusters, comparing observed and expected frequencies in each cell (expected frequencies in parentheses)

Cluster	Non-clinical sample (*N* = 637)	Clinical sample (*N* = 172)
1	104 (82)**	0 (22)***
2	108 (85)**	0 (23)***
3	42 (49)	20 (13)*
4	73 (65)	10 (18)*
5	83 (74)	11 (20)*
6	26 (38)*	22 (10)***
7	23 (69)***	64 (19)***
8	100 (88)	12 (24)**
9	78 (87)	33 (24)*

* *p* < .05; ** *p* < .01; *** *p* < .001

### Combining Low Use of Acceptance with Low Use of Cognitive Restructuring

As seen in Fig. 1, one of the clusters (Cluster 6) was characterized by consistently low scores specifically on acceptance in combination with about average scores on cognitive restructuring, whereas another cluster (cluster 7) showed consistently low scores on both acceptance and cognitive restructuring. Providing partial support for the “no-strategies-is-worse” hypothesis, Cluster 7 scored significantly lower than Cluster 6 on positive emotionality (see Table 3). However, there were no significant differences between the clusters on negative emotionality (Table 5) or life quality (Table 6), and both clusters showed similar over-representation in the clinical sample and under-representation in the non-clinical sample (see Table 7).

## Discussion

The primary purpose of the present study was to test the hypothesis that the use of distractive strategies is adaptive when combined with acceptance, and maladaptive when combined with avoidance. A secondary purpose was to compare alternative hypotheses with regard to the effects of combining high use, and low use, respectively, of acceptance with high use of cognitive restructuring strategies.

The study clearly supported the hypothesis that distractive strategies are adaptive when combined with acceptance, and maladaptive when combined with avoidance. This is interesting in view of the ambiguous status of distraction strategies within cognitive behavioral therapies, where distraction (e.g., Gratz 2010; Linehan 1993b) are taught as valid strategies for regulating aversive emotions in some protocols, and are seen as counter-productive in others (e.g. Craske and Barlow 2008). The results of the present study suggest that the function of distraction strategies depend upon whether distraction is used primarily as a way of avoiding aversive experiences, or used merely to redirect attention towards something else for a short period of time, merely postponing one’s willingness to enter into contact with the avoided emotion.

Methodologically, it may be noted that the hypothesis on distraction could be tested because the cluster analysis resulted in two clusters with almost equally high scores on Distractive Refocusing, combined with almost opposite profiles on the acceptance variables. The secondary hypotheses could not be equally well tested because the cluster analysis did not produce all cluster profiles required for this. In particular, although the analysis produced one cluster with generally high scores both on acceptance and cognitive restructuring strategies, and another cluster with high scores on acceptance strategies and low scores on cognitive restructuring strategies, no cluster was found with generally high scores on cognitive restructuring combined with low scores on acceptance strategies. This means that, although the additive-interactive hypothesis could be rejected (because the cluster with high scores on both cognitive restructuring and acceptance was not associated with higher well-being than the cluster with high scores merely on acceptance), the results were consistent with both of the other hypotheses – that is, the equifinality hypothesis (i.e., acceptance and cognitive change strategies achieve similar outcomes), and the “acceptance-is-essential hypothesis”.

There are important limitations to the present study that should be taken into consideration when interpreting the results. First, the study is based upon the factor structure identified only in a previous study by the same authors (Wolgast et al. 2012), and the present study makes use of the same data as in the previous study. It remains to see whether the results generalize to other populations. Furthermore, the design is cross-sectional, which renders causal conclusion regarding the relationship between the examined strategies and the criterion variables impossible to draw. As an example, we cannot know if a particular profile of regulatory strategies leads to emotional distress or whether it is high levels of emotional distress leads one to adopt a particular approach to emotion regulation. Furthermore, the study is based entirely upon data from self-report questionnaires, which make the results susceptible to threats from mono-method biases. A particular limitation tied to the use of self-report measures is that the validity of the data is dependent upon the degree to which the participants are consciously aware of their habitual use of different strategies and traits. With this in mind, a recommendation for future research would be to study the different forms of distraction strategies experimentally as well as to capture not only explicit, but also implicit, automatic or unconscious distraction strategies. It would also be interesting to study whether the effects of distraction strategies varies depending on for example what emotion or experience we distract ourselves from and if you examine long term or short term consequences.

Despite these limitations, we believe that the present study provides interesting empirical contributions to the ongoing discussion regarding the role of distraction in emotion regulation. It also illustrates the advantages of applying a person-oriented approach to the study of emotion regulation which, instead of focusing on variables, studies different ways of combining several emotion regulatory strategies at the level of the individual.
